# Carbonic anhydrase I (CA1) is involved in the process of bone formation and is susceptible to ankylosing spondylitis

**DOI:** 10.1186/ar3929

**Published:** 2012-07-27

**Authors:** Xiaotian Chang, Yabing Zheng, Qingrui Yang, Lin Wang, Jihong Pan, Yifang Xia, Xinfeng Yan, Jinxiang Han

**Affiliations:** 1 Medical Research Center of Shandong Qianfoshan Hospital, Shandong University. Jingshi Road 16766, Jinan, Shandong, 250014 P. R. China; 2 Shandong Academy of Medical Sciences. Jingshi Road 18877, Jinan, Shandong, 250062 P. R. China; 3 Department of Rheumatology, Provincial Hospital of Shandong, Shandong University, Jingwuweiqi Road 324, Jinan, Shandong, 250021 P. R. China; 4 Orthopedic Surgery Center of Shandong Qianfoshan Hospital, Shandong University, Jingshi Road 16766, Jinan, Shandong, 250014 P. R. China

## Abstract

**Introduction:**

Ankylosing spondylitis (AS) is characterized by abnormal bone formation in the spine and the sacroiliac joints. *In vitro *assays demonstrate that carbonic anhydrase I (CA1) promotes calcium precipitation. This study investigated the function of CA1 for bio-mineralization and determined if common polymorphisms in the CA1 gene might contribute to AS risk.

**Methods:**

Calcification was induced in Saos-2 cells, a human osteosarcoma cell line, with ascorbic acid and β-glycerophosphate. Calcification was determined by Alizarin Red-S (AR-S) staining. Expressions of CA1, alkaline phosphatase (ALP), bone sialoprotein (BSP), osteocalcin (OCN), osterix (OSX) and runt-related transcription factor-2 (Runx2) were determined by real-time PCR and western blotting. The cells were also treated with acetazolamide, an anti-carbonic anhydrase drug. Genotyping was performed using Illumina VeraCode microarray in a case-control study including 51 AS patients, 267 rheumatoid arthritis (RA) patients and 160 healthy controls. The result was confirmed by Taqman assay, including 258 AS patients, 288 RA patients and 288 healthy controls.

**Results:**

Following the induction of calcification, Saos-2 cells produced large amounts of calcium-rich deposits. Increased transcriptions of CA1, ALP, BSP, OCN, OSX and Runx2, essential genes for ossification, were detected in the cultured cells. Following treatmen with acetazolamide, the expression of CA1 obviously declined and mineralized nodule formation was also decreased. Illumina microarray indicates that SNP at rs7841425 also showed significant differences in allelic frequency (*P *= 0.01396) and genotypic frequency (*P *= 0.005902) between AS cases and controls. In addition, SNP at rs7827474 showed significant differences in allelic frequency (*P *= 5.83E-04) and genotypic frequency (*P *= 0.000186) between RA cases and controls (*P *values were adjusted to multiple comparisons). The Taqman assay revealed that rs725605 demonstrated statistically significant evidence of allele frequency (*P *= 0.022307) and gene frequency (*P *= 0.007731) for association with AS. This SNP did not show significant differences in allelic frequencies and gene frequencies between RA patients and controls.

**Conclusions:**

CA1 may play an essential role in bio-mineralization and new bone formation. The gene encoding CA1 is susceptible to AS.

## Introduction

Abnormal new bone formation and bone resorption are the most distinctive features of ankylosing spondylitis (AS) [1-3]. Histopathological experiments demonstrated that severe forms of AS significantly correlate with villous chronic synovitis, including obliterating vasculitis, fibrosclerosis, necrosis and calcification of disintegrated synovial structures [4]. Using a proteomic approach, we previously screened novel AS-specific proteins by simultaneously comparing the expression profiles of synovial membranes from patients with AS, rheumatoid arthritis (RA) and osteoarthritis (OA). The proteomic study revealed significantly increased expression of carbonic anhydrase I (CA1) in the synovial membrane of AS patients compared with those of RA and OA patients. Immunohistochemistry and western blot analysis confirmed the above findings [5]. CA1 is a member of the carbonic anhydrase (CA) family, which catalyzes the reversible hydration and dehydration reactions of CO_2_/H_2_CO_3 _[6]. *In vitro *assays demonstrated that CA1 not only enhances the hydration reaction but also promotes the formation of CaCO_3 _[7,8]. Calcium salt precipitation is an important step in bone formation. Thus, the increased CA1 expression in the synovium of AS patients may lead to improper mineralization by accelerating calcium salt deposition [5]. However, no direct data support the involvement of CA1 in bio-mineralization and bone formation in cells and tissues.

In this study, we selected Saos-2 cells as a convenient model of osteoblastic mineralization to investigate how CA1 is involved in bio-mineralization and ossification *in vivo*. The cell line was induced with β-glycerophosphate (β-GP) and ascorbic acid (AA). We determined the process of calcification by examining the expression of alkaline phosphatase (ALP), bone sialoprotein (BSP), osteocalcin (OCN), osterix (OSX), and runt-related gene 2 (Runx2), protein markers for ossification. We also investigated the expression of CA1 in the cultured cells. Furthermore, we observed the calcification in the cultured cells in the presence of acetazolamide, an anti-carbonic anhydrase drug to CA1. In addition, we determined if common polymorphisms in the CA1 encoding gene are susceptible to AS in the Chinese population using the Illumina GoldenGate assay and Taqman genotyping methods. The genotyping was performed in a case-control study using a cohort of patients with rheumatoid arthritis (RA) and healthy individuals as controls. To our knowledge, this is the first study to examine this potential association.

## Methods

### Cell culture and induction of bio-mineralization

Saos-2 cells were cultured in McCoy's 5A medium (Gibco, Grand Island, New York, USA) supplemented with 100 U/ml penicillin, 100 mg/ml streptomycin (Gibco) and 15% (v/v) fetal bovine serum (Gibco). The cells were cultured either in basic cell culture medium or in osteogenic medium (OM) supplemented with 50 mg/ml AA (Sigma-Aldrich, St. Louis, Missouri, USA) and 7.5 mM β-GP (Sigma). The protocol was based on the study by Thouverey [9].

### Alizarin Red-S staining and quantifying bio-mineralization

Cell cultures were washed with Phosphate buffered saline buffer (PBS) and stained with 0.5% (w/v) Alizarin Red-S (Sigma-Aldrich ) in PBS (pH = 5.0) for 30 minutes at room temperature. After washing four times with PBS, the stained cells were photographed. The cultured cells were then de-stained with 10% (w/v) cetylpyridinium chloride (Sigma-Aldrich, ) in PBS (pH = 7.0) for 60 minutes at room temperature. The AR-S concentration was determined by measuring the absorbance at 562 nm. The protocol was based on previously published studies [9-11].

### The effects of acetazolamide on bio-mineralization

To verify the effect of CA1 expression on bio-mineralization, acetazolamide, a chemical inhibitor of CA, was used to treat Saos-2 cells. Acetazolamide (Sigma) was dissolved in dimethyl sulfoxide (DMSO, Solabio) at a concentration of 0.05% and was added to cell cultures that were grown to 70% confluence in OM. The cultures were grown in the presence of acetazolamide at a final concentration of 100 μM for nine days. Saos-2 cells were also cultured in OM with 0.05% DMSO as a control. The protocol was based on a study by Hall *et al*. [12].

### Cell proliferation assay

To evaluate the proliferative capacity of Saos-2 cells in OM or in the presence of acetazolamide, the cells were seeded onto 96-well culture plates and incubated until they reached 70% confluence. The cultures were then incubated with OM in the presence of 10 μM, 100 μM or 1 mM acetazolamide for three, six and nine days. Cell proliferation was assessed using the MTT (3-(4, 5-dimethyl-2-thiazolyl)-2, 5-diphenyl-2H-tetrazolium bromide) assay. Aliquots of 20 μl of MTT reagent of MTT Cell Proliferation Assay Kit (ATCC, Manassas, Virginia, USA) at a concentration of 5 mg/ml were added to the cell cultures. After four hours of treatment, 150 μl of DMSO was added to the culture and slightly mixed for 10 minutes. The absorbance was measured at 490 nm with a spectrophotometer.

### RNA isolation and real-time PCR

Total RNA was extracted from the cultured Saos-2 cells using a total RNA Isolation Kit (Omega Bio-Tek, Norcross, Georgia, USA) according to the protocol of the manufacturer. Concentrations of total RNA were determined with a spectrophotometer. The cDNA was prepared with 500 ng of total RNA from each sample using oligo-dT primers and the PrimeScriptTM RT-PCR Kit (TaKaRa, Ostsu, Shiga, Japan). A quantitative PCR analysis was performed with a LightCycler 480 thermocycler (Roche, Basel, Switzerland.). Taqman real-time PCR was conducted in 10 μl reaction mixtures containing 5 μl of SYBR Green (ToYoBo, Osaka, Japan), 1 μl of each primer, 1 μl of cDNA and 2 μl of H_2_O. The optimized thermal cycling program was as follows: step one, 95ºC for 180 seconds; step two, 95ºC for 10 seconds, 59ºC for 10 seconds and 72ºC for 25 seconds (45 cycles); and step three, a melting curve analysis from 59°C to 95ºC in 0.5ºC increments. The specificity of the individual amplification reactions was assessed by examining the melting curves to confirm the presence of a single gene-specific peak with the characteristic melting temperature of the expected product. The primers for ALP, BSP, OCN, OSX, Runx2 and CA1 are listed in Table 1. The PCR results were standardized to the level of glyceraldehyde 3-phosphate dehydrogenase (GAPDH) expression. For each sample, two reactions were performed at the same time. One reaction was performed to determine the mRNA level of the target gene and the second reaction was performed to determine the level of GAPDH. The experiment was performed in triplicate and the PCR products were confirmed by a melting curve analysis. The relative expression of mRNA was calculated using the comparative threshold cycle (Ct) method according to the following formula: Ratio = 2-ΔΔCt = 2ΔCt (sample), where ΔCt = Ct of target genes-Ct of the endogenous control gene (GAPDH). The relative target gene expression was normalized in comparison to the GAPDH mRNA. The copy numbers of the targeted mRNA in the samples were calculated automatically by the data analysis software. Levels of the transcripts are expressed as the median and range. Statistical differences were assessed using the Mann-Whitney U-test; *P *<0.05 was considered statistically significant.

**Table 1:** The primer sequences for real-time PCR.

gene	Forward and Reverse Primers
ALP	5'-TGGACCTCGTTGACACC-3' 5'-TCCTGTTCAGCTCGTACT -3'
BSP	5'-ATACAGGGTTAGCTGCAATC-3' 5'- TCCATTGTCTCCTCCGC -3'
OCN	5'-GGCGCTACCTGTATCAATG-3' 5'-AGAGCGACACCCTAGAC -3'
OSX	5'-CCACCTACCCATCTGACT-3' 5'-GTTTGGCTCCACCACTC -3'
Runx2	5'-TATTGGTCCTAAGGGAGACATC-3' 5'-TAAAGCGAATGGGCATGTT -3'
CA1	5'-GCTACAGGCTCTTTCAGTT-3' 5'-GACTCCATCCACTGTATGTT -3'
GAPDH	5'-ACCACAGTCCATGCCATCAC-3' 5'-TCCACCACCCTGTTGCTGTA -3'

### Western blot analysis

Cultured cells and animal tissues were homogenized in RIPA buffer (Beyotime, Shanghai, China) supplemented with protease and phosphatase inhibitors on ice and centrifuged at 16000 × g for five minutes at 4°C. The supernatant was collected and the protein concentration was determined using a BCA Protein Assay Kit (Beyotime). Thirty micrograms of total protein were separated on a 12% SDS-polyacrylamide gel and transferred onto a PVDF membrane. The membrane was rinsed with wash solution and incubated with anti-human CA1 antibody (Santa Cruz, Santa Cruz, California, USA) at a dilution of 1:500 overnight at 4°C. The antibody was prepared by immunizing a mouse with a peptide comprising CA1 amino acids 33 to 80, which are mapped near the N-terminus. The manufacturer confirmed no cross-reactivity with other carbonic anhydrases. Immunosignals were visualized with a Protein Detector BCIP/NBT Western Blotting Kit (Beyotime) following the manufacturer's instructions. The quantification was conducted using ImageQuant 5.2 software. A separate membrane prepared in the same manner was probed with an anti-GAPDH antibody (Santa Cruz) to normalize the sample loading.

### Sample preparation and genomic DNA isolation

Peripheral blood samples were collected from patients with RA and AS. The diagnosis of RA was made according to the criteria of the American College of Rheumatology [13]. The RA patients had high levels of C-reactive protein (30 to 100 mg/L, mean 24 mg/L), anti-cyclic citrullinated protein antibodies (anti-CCP; 300 to 3,000 U/ml) and rheumatoid factor (RF; 160 to 2,560 U/ml). AS patients had an average disease duration of seven years and were positive for HLA-B27 antigen. Their symptoms were consistent with the modified New York criteria for AS. Patients were selected from the same population living in the Shandong area of Northern China. They did not have any personal or family history of serious illness. Control individuals were from the same geographical area. Both patients and healthy controls gave their written consent to participate in the study and to allow their biological samples to be genetically analyzed. The Ethical Committee of the Shandong Academy of Medicinal Sciences approved this study.

Blood samples were put into Monovette tubes containing 3.8% sodium citrate. Genomic DNA was extracted from whole blood samples with the Omega E-Z 96 Blood DNA kit (Omega) according to the manufacturer's protocol. DNA samples were dissolved in deionized water. The concentration and quality of DNA samples were measured using a spectrophotometer at 260 nm (A260) and 280 nm (A280). An A260/A280 ratio of 1.7 to 1.9 was considered to be highly pure DNA. Electrophoresis was also used to examine the quality and integrity of DNA.

### SNP selection and genotyping

Tag SNPs across the CA1 were determined by searching the HapMap database. The tag SNPs were selected on the basis of linkage disequilibrium patterns observed in the Han Chinese in Beijing (CHB) samples that were genotyped as a part of the International HapMap Project. Only SNPs with a minor allele frequency of greater than 5% with a pair-wise r^2 ^≥ 0.8 in the HapMap were considered. SNPs that were in exons and UTRs, as well as promoters and introns within 500 bp of exons were also selected. Candidate SNPs were submitted to Illumina for a design score. The Illumina Assay Design Tool filtered out SNPs that were not suitable for the Illumina platform. Finally, thirteen SNPs with a design score of '1' were selected; they span 23,000 bases of the chromosome. These SNPs included one tagSNP, one coding SNP, one SNP at the 5'UTR, nine SNPs in the introns and one SNP in the promoter region. These SNP sites and locations are described in Table 2.

**Table 2:** SNP information.

SNP ID	chr position	function	allele	amino acid
rs7827474	86241688	intron	C/G	
rs3808540	86244170	intron	C/T	
rs11774422	86244588	intron	A/G	
rs7011926	86245233	intron	A/G	
rs34456327	86245492	intron	-/G	
rs7821248	86245776	missense	A	Glu [E]
rs13276893	86250947	intron	C/T	
rs2220972	86253296	intron	A/G	
rs34282382	86264002	5' UTR	-/G	
rs7840971	86264516	intron	C/T	
rs7841304	86264767	intron	C/T	
rs7818073	86264811	intron	A/T	
rs7841425	86264813	intron	A/C	

We performed genotyping using a custom-designed Illumina 96-SNP VeraCode microarray (Illumina). Peripheral blood samples were collected from patients with RA (n = 266, 183 women) and AS (n = 51, 10 women). RA patients had a mean age of 51.7 years, while AS patients had a mean age of 35.9 years. A total of 163 (60 women) healthy individuals with a mean age of 48.0 years were blood donors. Blood samples were put into Monovette tubes containing 3.8% sodium citrate. The genotyping was conducted with the BeadXpress Reader using the Illumina VeraCode GoldenGate Assay kit. A total of 500 ng of sample DNA were used per assay. Genotype clustering and calling were performed using BeadStudio software (Illumina). This work was completed at the Beijing Institute of Genomics.

To confirm the above results, tag SNP rs725605 was genotyped using TaqMan SNP assays in a cohort of 288 patients with RA (209 women), 258 patients with AS (85 women) and 288 healthy controls (132 women). These people are different than the people used in the experiments of Illumina microarray assay. RA patients had a mean age of 52.2 years, AS patients had a mean age of 31.6 years and the healthy individuals had a mean age of 63.5 years. Assays were run on a LightCycler II 480 Instrument (Roche) and evaluated according to the manufacturer's instructions. Reactions were carried out in a total volume of 10 μl using the following amplification protocol: denaturation at 95°C for 10 minutes, followed by 40 cycles of denaturation at 92°C for 15 seconds and finishing with annealing and extension at 60°C for 1 minute. The genotype of each sample was determined by measuring allele-specific fluorescence using SDS 2.3 software for allelic discrimination (Roche). Duplicate samples and negative controls were included to check the accuracy of genotyping.

Genotyping quality was examined by a detailed quality control procedure consisting of >95% successful call rate, duplicate calling of genotypes, internal positive control samples and Hardy-Weinberg Equilibrium (HWE) testing. SNPs were analyzed for association by comparing the MAF (Minor Allele Frequency) between cases and controls. Dominant and recessive models were considered with respect to the minor allele. Associations of SNPs with the diseases were evaluated using odds ratios (OR) with 95% confidence intervals (CI). Fisher's exact test was used for comparison between categorical variables. *P *values less than 0.05 were considered statistically significant. Genotypic associations were assessed using software Plink v1.07 and SHEsis [14,15]. Multiple-test correction including genomic-control correction, Bonferroni single-step correction, Holm step-down correction and Sidak single-step correction were performed by Plink v1.07. Linkage disequlibrium (LD), coefficient (D' and r^2^) and haplotype were estimated by software Haploview 4.2 [14].

### Statistical analyses

Statistical analyses of the data were performed using SPSS V.16 software (SPSS, Chicago, Illinois, USA). The multiple comparisons were conducted using analysis of variance (ANOVA). T-test was used to assess statistical differences of two groups. *P *values less than 0.05 were considered significant.

## Results

### Bio-mineralization in Saos-2 cells incubated with OM and acetazolamide

**Figure 1:**
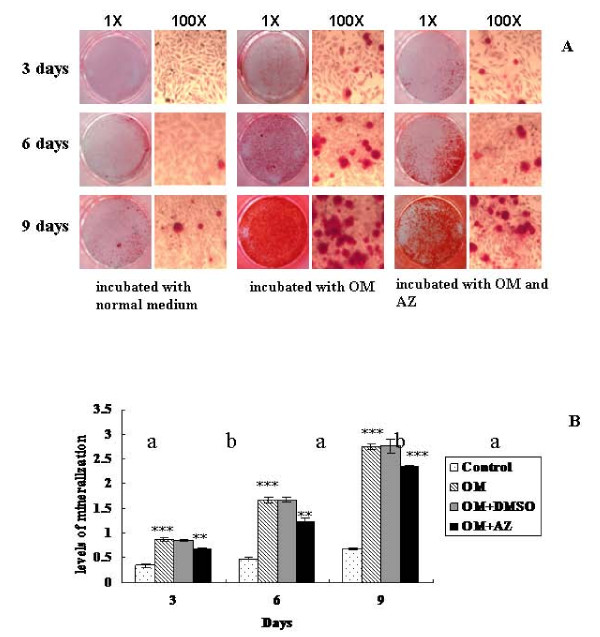
**Measuring bio-mineralization in Saos-2 cells cultured with OM and acetazolamide (AZ)**. The Saos-2 cells were incubated with normal medium, osteogenic medium (OM), and OM supplemented with 100 μM of AZ. **A**. The cell cultures were stained with AR-S to detect calcium nodules and photographed at the original magnification and 100X magnification. **B**. The cell cultures were stained with cetylpyridinium chloride and quantified by measuring its absorbance at 562 nm. This experiment was repeated three times. ** = *P *< 0.01, *** = *P *< 0.001.

A large amount of calcium deposits was observed in the cultured cells when the incubation was conducted in OM for six and nine days (Figure 1A). The absorbance of cetylpyridinium chloride was 3.46-fold higher in Saos-2 cells that were incubated with OM than in the cells that were cultured without induction for six days (*P *= 4.63*10-5). The absorbance was 4.06-fold higher after nine days than that of the control (*P *= 3.92*10-7) (Figure 1B). The formation of calcium nodules was significantly decreased in Saos-2 cells when acetazolamide was included in the OM compared with the cells that were stimulated with OM alone (Figure 1A). The absorbance of cetylpyridinium chloride was also considerably decreased by 1.28-fold in Saos-2 cells that were treated with acetazolamide for three days (*P *= 0.0003), 1.36-fold after six days (*P *= 0.0008) and 1.26-fold after nine days (*P *= 0.0001) when compared with cells that were cultured with OM alone (Figure 1B).

### Cell proliferation of Saos-2 cells incubated with OM and acetazolamide

**Figure 2:**
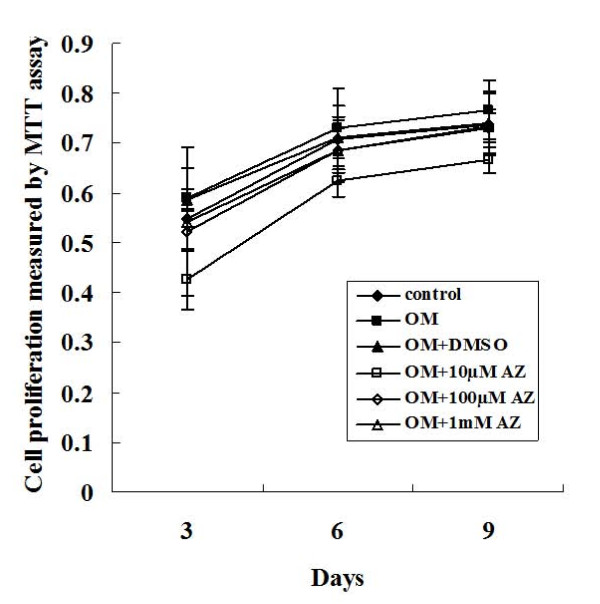
**Measuring cell proliferation by the MTT assay**. Saos-2 cells were incubated in (1) normal culture medium, (2) osteogenic medium, (3) osteogenic medium supplemented with DMSO, and (4) osteogenic medium supplemented with 10 μM, 100 μM and 1 mM of acetazolamide (AZ). The results of the MTT assay indicated that the cell proliferation was not significantly affected by osteogenic medium, DMSO or 100 μM AZ. This experiment was repeated three times and similar results were obtained. The error bars represent the standard deviation from five replicate measurements. DMSO, dimethyl sulfoxide; MTT, 3-(4, 5-dimethyl-2-thiazolyl)-2, 5-diphenyl-2H-tetrazolium bromide; OM, osteogenic medium.

Saos-2 cells were incubated with OM in the absence or presence of acetazolamide at concentrations of 10 μM, 100 μM and 1 mM for three, six and nine days. The number of living cells was slightly increased in Saos-2 cells that were cultured in OM compared with the control group in normal medium for three, six and nine days, but there were no significant changes between the two cultures. The proliferation of Saos-2 cells was not significantly different between cells that were incubated with OM in the presence of 100 μM acetazolamide for three days, six days and nine days. When Saos-2 cells were incubated with OM in the presence of 1 mM acetazolamide, cell proliferation was significantly decreased on the third day (*P *= 0.03), the sixth day (*P *= 0.023) and the ninth day (*P *= 0.035) of the incubation when compared with cells that were cultured with OM alone. Therefore, 100 μM was considered to be the highest non-toxic concentration of acetazolamide and was used in the subsequent assays. The results are depicted in Figure 2.

### Expression of osteoblast-related genes and CA1 in Saos-2 cells during bio-mineralization

**Figure 3:**
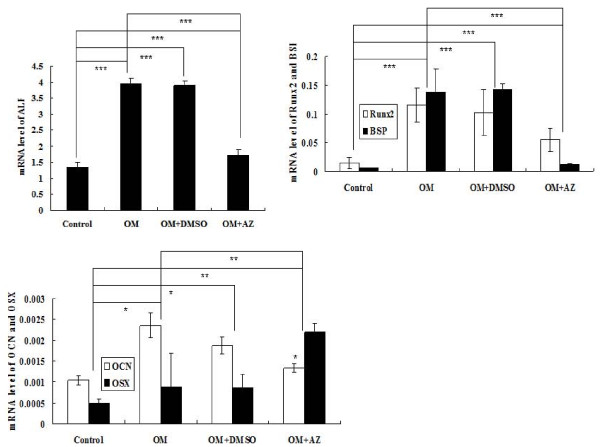
**Quantification of the expression of ALP, BSP, OCN, OSX and Runx2 by real-time PCR**. Real-time PCR was used to measure the mRNA levels of ALP, BSP, OCN, OSX and Runx2 mRNA in Saos-2 cells incubated with OM. The expression was normalized to the expression of GAPDH. The results indicate that OM can stimulate transcription of these osteoblast-related genes in Saos-2 cells than in than cells without stimulation. The also indicates that AZ can significantly decrease the expressions of these osteoblast-related genes except OSX in Saos-2 cells compared to that in OM alone. This experiment was repeated three times and similar results were obtained. * = *P *< 0.05, ** = *P *< 0.01, *** = *P *< 0.001. OM, osteogenic medium.

The mRNA level of Runx2 was 8.05-fold (*P *= 3.57*10-7) higher in the stimulated cells than in cells without stimulation, ALP increased 2.91-fold (*P *= 0.000929), BSP increased 27.28-fold (*P *= 5.63*10^-5^), OCN increased 2.48-fold (*P *= 0.04) and OSX increased 1.8-fold (*P *= 0.002), when Saos-2 cells were incubated in OM for nine days. After nine days of treatment with 100 μM acetazolamide in OM, the expression levels of ALP (*P *= 8.71*10^-5^), BSP (*P *= 0.001), OCN (*P *= 0.007) and Runx2 (*P *= 5.74*10^-5^) were significantly decreased in Saos-2 cells compared to their levels in OM alone, except for OSX (*P *= 0.026). These results are shown in Figure 3.

**Figure 4:**
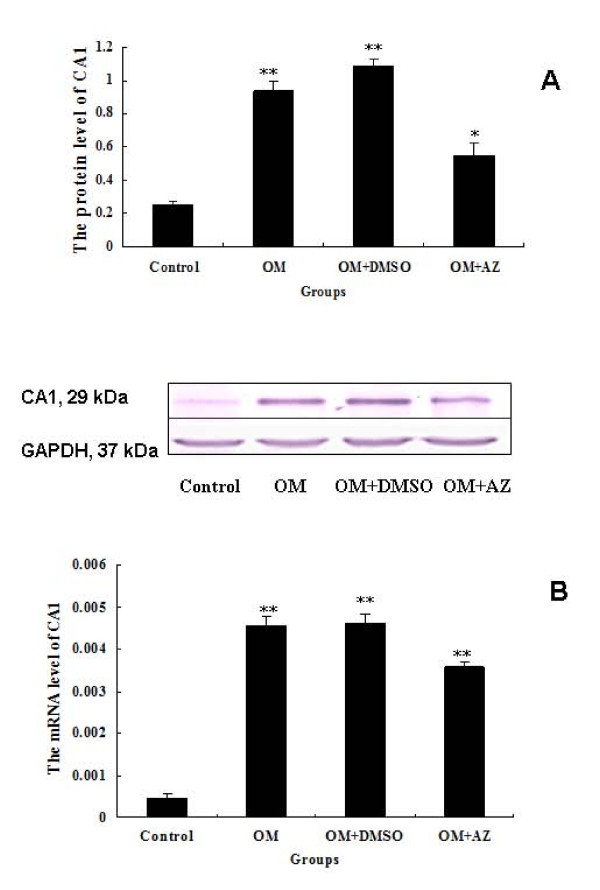
**Expression of CA1 in Saos-2 cells**. The expression of CA1 in Saos-2 cells was determined by (**A**) western blotting and (**B**) real-time PCR. The expression levels were normalized to the expression of GAPDH. The transcription and translation of CA1 were significantly increased in Saos-2 cells cultured in the presence of OM, but decreased when the cells were incubated with 100 μM acetazolamide (AZ). This experiment was repeated three times and similar results were obtained. * *P *< 0.05, ** = *P *< 0.01, *** = *P *< 0.001. OM, osteogenic medium.

The transcription (*P *= 0.007) and translation (*P *= 0.009) of CA1 in the stimulated cells were significantly affected in a time-dependent manner for nine day's culture compared with the controls. After treatment with acetazolamide, CA1 transcription (*P *= 0.004) and translation (*P *= 0.012) were significantly decreased compared with the cells cultured in OM alone. These results are presented in Figure 4.

### Single SNP analysis

We genotyped twenty-nine SNPs in the CA1 gene. All SNPs yielded usable genotyping data and the study sample success rate was 99% [See Additional file 1]. The Illumina microarray data has been submitted to NCBI Gene Expression Omnibus (GEO), a public functional genomics data repository supporting MIAME-compliant data submissions. The record was approved and assigned GEO accession number (GSE39428). Differences in the allele and genotype frequencies between cases and controls were compared. Allelic frequency and genotype frequencies of all SNPs were in Hardy-Weinberg equilibrium in controls. SNP at rs34282382 showed significant differences in allelic frequency and genotypic frequency in the AS cohort. SNP at rs7841425 also showed significant differences in allelic frequency and genotypic frequency between AS cases and controls. Following multiple-test correction, rs7841425 still had a significant difference in allelic frequency and genotypic frequency. In addition, SNP at rs7827474 had a significant difference in allelic frequency and genotypic frequency between RA cases and controls. After multiple-test correction, rs7827474 still had a significant difference in allelic frequency and genotypic frequency between RA cases and controls, indicating that the SNP is significantly associated with an increased risk of RA. Other SNPs located in the CA1 gene were not significantly different in allelic or genotypic frequencies between the RA or AS patients and controls. Tables 3 and 4 contain the allele frequencies, genotype frequencies and the unadjusted and adjusted *P*-values after the statistical analysis of these genotyped SNPs. We also performed multivariate logistic regression analysis using software Plink v1.07 to test the correlations between the SNP and RA and AS. The *P *values for rs7827474 and rs7841425 after logistic regression are also shown in Tables 3 and 4.

**Table 3:** Allele and genotype frequencies in a case control cohort of patients with AS.

dbSNP identity	Allele/ Geno type	Number of patients with RA (%)	Number of controls (%)	*P *value	*P *value after multivariate logistic regression	*P *value after multiple testing correction (Bonferroni method)	Odds Ratio (%95 CI)	HWE test for case (df = 1)	HWE test for control (df = 1)
rs7827474	C	1(0.010)	1(0.003)	0.395319			3.138614 (0.194542~50.636475)		
	G	101(0.990)	317(0.997)						
	CG	1(0.020)	1(0.006)	0.394186				0.943634	0.968272
	GG	50(0.980)	158(0.994)						
									
rs3808540*	A	64(0.627)	196(0.613)	0.786888			1.065521 (0.672573~1.688048}		
	G	38(0.373)	124(0.388)						
	AA	19(0.373)	64(0.400)	0.475302				0.518298	0.185552
	AG	26(0.510)	68(0.425)						
	GG	6(0.118)	28(0.175)						
									
rs11774422	A	0(0.000)	1(0.003)	0.571929					
	G	102(1.000)	319(0.997)						
	AG	0(0.000)	1(0.006)	0.571472				1	0.968372
	GG	51(1.000)	159(0.994)						
									
rs34456327	A	98(1.000)	312(1.000)						
	AA	49(1.000)	156(1.000)						
									
rs7821248	A	0(0.000)	1(0.003)	0.575955					
	G	98(1.000)	313(0.997)						
	AG	0(0.000)	1(0.006)	0.57549				1	0.968069
	GG	49(1.000)	156(0.994)						
									
									
rs13276893	A	69(0.704)	218(0.717)	0.804077			0.938627 (0.569058~1.548209)		
	G	29(0.296)	86(0.283)						
	AA	22(0.449)	77(0.507)	0.475953				0.116312	0.641532
	AG	25(0.510)	64(0.421)						
	GG	2(0.041)	11(0.072)						
									
									
rs2220972	G	102(1.000)	320(1.000)						
	GG	51(1.000)	160(1.000)						
									
rs1032551	C	30(0.306)	110(0.374)	0.2236			0.737968 (0.451984~1.204903)		
	G	68(0.694)	184(0.626)						
	CC	0(0.000)	13(0.088)	0.096659					
	CG	30(0.612)	84(0.571)						
	GG	19(0.388)	50(0.340)						
									
rs34282382	A	102(1.000)	298(0.949)	0.020115					
	C	0(0.000)	6(0.051)						
	AA	51(1.000)	141(0.898)	0.017687				1	0.501138
	AC	0(0.000)	16(0.102)						
									
rs7840971	A	0(0.000)	1(0.003)	0.571929					
	G	102(1.000)	319(0.997)						
	AG	0(0.000)	1(0.006)	0.571472				1	0.968372
	GG	51(1.000)	159(0.994)						
									
rs7841304	A	0(0.000)	1(0.003)	0.576716					
	G	92(1.000)	295(0.997)						
	AG	0(0.000)	1(0.007)	0.576223				1	0.967107
	GG	46(1.000)	147(0.993)						
									
rs7818073	T	102(1.000)	320(1.000)						
	TT	51(1.000)	160(1.000)						
									
rs7841425	A	15(0.153)	91(0.318)	0.001611	0.002234	0.01396	0.387263 (0.211775~0.708169}		
	C	83(0.847)	195(0.682)						
	AA	0(0.000)	17(0.119)	0.007879		0.005902		0.205909	0.330884
	AC	15(0.306)	57(0.399)						
	CC	34(0.694)	69(0.483)						

CI, confidence interval; HWE, Hardy-Weinberg equilibrium.

**Table 4:** Allele and genotype frequencies in a case control cohort of patients with RA.

dbSNP identity	Allele/ Geno type	Number of patients with RA (%)	Number of controls (%)	*P *value	*P *value after multivariate logistic regression	*P *value after multiple testing correction (Bonferroni method)	Odds Ratio (%95 CI)	HWE test for case (df = 1)	HWE test for control (df = 1)
rs7827474	C	31(0.059)	1(0.003)	3.68E-05	3.31E-03	5.83E-04	20.014257 (2.718505~147.349579)		
	G	491(0.941)	317(0.997)						
	CC	1(0.004)	0(0.000	0.0002		0.000186		0.929804	0.968272
	CG	29(0.111)	1(0.006)						
	GG	231(0.885)	158(0.994)						
									
rs3808540*	A	307(0.590)	196(0.613)	0.525421			0.911852 (0.685840~1.212343)		
	G	213(0.410)	124(0.388)						
	AA	91(0.350)	64(0.400)	0.506516				0.923195	0.185552
	AG	125(0.481)	68(0.425)						
	GG	44(0.169)	28(0.175)						
									
rs11774422	A	4(0.008)	1(0.003)	0.414051			2.425855 (0.269936~21.800669)		
	G	526(0.992)	319(0.997)						
	AG	4(0.015)	1(0.006)	0.412665				0.901484	0.968372
	GG	261(0.985)	159(0.994)						
									
rs7011926	A	287(0.542)	172(0.541)	0.985804			1.002536 (0.758458~1.325161)		
	G	243(0.458)	146(0.459)						
	AA	77(0.291)	55(0.346)	0.079064				0.86122	0.006761
	AG	133(0.502)	62(0.390)						
	GG	55(0.208)	42(0.264)						
									
rs34456327	A	7(0.013)	312(1.000)	0.040401					
	G	7(0.013)	0(0.000)						
	AA	257(0.981)	156(1.000)	0.221769				3.33E-15	1
	AG	3(0.011)	0(0.000)						
	GG	2(0.008)	0(0.000)						
									
rs7821248	A	2(0.004)	1(0.003)	0.874655			1.213178 (0.109552~13.434785)		
	G	516(0.996)	313(0.997)						
	AA	1(0.004)	0(0.000)	0.323685				5.55E-16	0.968069
	AG	0(0.000)	1(0.006)						
	GG	258(0.996)	156(0.994)						
									
rs13276893	A	359(0.730)	218(0.717)	0.699646			1.064841 (0.773866~1.465222)		
	G	133(0.270)	86(0.283)						
	AA	122(0.496)	77(0.507)	0.238757				0.003729	0.641532
	AG	115(0.467)	64(0.421)						
	GG	9(0.037)	11(0.072)						
									
rs2220972	A	1(0.002)	0(0.000)	0.438632					
	G	533(0.998)	320(1.000)						
	AG	1(0.004)	0(0.000)	0.438364				0.975544	1
	GG	266(0.996)	160(1.000)						
									
rs34282382	A	502(0.954)	298(0.949)	0.725735			1.123042 (0.587088~2.148271)		
	C	24(0.046)	16(0.051)						
	AA	240(0.913)	141(0.898)	0.612061				0.521732	0.501138
	AC	22(0.084)	16(0.102)						
	CC	1(0.004)	0(0.000)						
									
rs7840971	A	5(0.009)	1(0.003)	0.285353			3.049713 (0.354681~26.222826)		
	G	523(0.991)	319(0.997)						
	AA	2(0.008)	0(0.000)	0.511044				3.00E-15	0.968372
	AG	1(0.004)	1(0.006)						
	GG	261(0.989)	159(0.994)						
									
rs7841304	A	2(0.004)	1(0.003)	0.887385			1.189516 (0.107391~13.175623)		
	G	496(0.996)	295(0.997)						
	AG	2(0.008)	1(0.007)	0.887172				0.949269	0.967107
	GG	247(0.992)	147(0.993)						
									
rs7818073	A	4(0.008)	0(0.000)	0.12006					
	T	528(0.992)	320(1.000)						
	AA	1(0.004)	0(0.000)	0.403133				4.44E-16	1
	AT	2(0.008)	0(0.000)						
	TT	263(0.989)	160(1.000)						
									
rs7841425	A	184(0.365)	91(0.318)	0.183639			1.232143 (0.905616~1.676401)		
	C	320(0.635)	195(0.682)						
	AA	46(0.183))	17(0.119)	0.250595				0.000748	0.330884
	AC	92(0.365)	57(0.399)						
	CC	114(0.452)	69(0.483)						

CI, confidence interval; HWE, Hardy-Weinberg equilibrium.

We performed additional genotyping for tag SNP rs725605 in an independent case-control study using the TaqMan method. Allelic frequencies and gene frequencies of the SNP did not deviate from HWE in both cases and the controls. The allele frequency and gene frequency for SNP rs725605 demonstrated statistically significant evidence for association with AS (*P *= 0.022307, OR (%95 CI) = 1.332797 (1.041573 to approximately 1.705446)). The gene frequency for the SNPs also demonstrated statistically significant evidence for association with AS (*P *= 0.007731). On the other hand, the SNPs rs725605 did not show significant difference in allelic frequencies (*P *= 0.367743) and gene frequencies (*P *= 0.610868) between RA patients and controls.

## Discussion

AA and β-GP are commonly employed to stimulate osteoblastic differentiation and induce *in vivo *mineralization [16,17]. Following the stimulation of AA and β-GP, a large amount of calcium-rich deposits was produced in Saos-2 cells, while the transcription levels of osteogenesis-related genes, including ALP, BSP, OCN, OSX and Runx2, were significantly increased. CA1 expression was also significantly increased in the stimulated cells. Following treatment with 100 uM acetazolamide, the expression of CA1 was obviously decreased in the cells, and the mRNA levels of ALP, BSP, OCN and Runx2 were dramatically decreased. Calcium nodule formation was also decreased in the treated Saos-2 cells. The inhibitor did not significantly reduce the cell proliferation. Thus, the decrease in mineralization was due to a direct effect on mineralization rather than decreased cell number. *In vitro *experiments indicated that CA1 catalyzes the generation of HCO_3_^- ^through the hydration of CO_2_, which then combines with Ca^2+ ^to form a CaCO_3 _precipitate [7,8]. Therefore, our study suggests that CA1 can promote the formation of calcium carbonate and calcification under physiological conditions. Mineralization is a key step for the formation, development, remodeling and repair of the osseous tissue [18,19]. The over-expression of CA1 very likely has an important role in ossification and new bone formation.

By genotyping twenty-nine SNPs located in the CA1 region, Illumina microassay demonstrated a significant association between rs34282382 and rs7841425 polymorphisms and susceptibility to AS in Chinese populations. Taqman genotyping confirmed the significant association between the CA1 polymorphism and susceptibility to AS risk. The above genotyping result suggests that the gene encoding CA1 is susceptible to AS.

SNP rs34282382 is located in the 5'-UTR of the CA1 gene. The 5'UTR of mRNA plays a critical role in translation regulation by influencing mRNA stability and translation efficiency. Functional elements in the 5'-UTR such as internal ribosome entry site (IRES), upstream ORF and iron responsive element (IRE) serve to fine tune protein expression in response to cellular requirements. Genetic variations such as mutations and SNPs in the 5'-UTR are associated with a number of human diseases and increased susceptibility to diseases. Riboswitches are non-protein coding RNA elements typically found in the 5'-UTR of mRNAs that utilize metabolite binding to control expression of their own transcript. The RNA-ligand interaction causes conformational changes in the RNA that direct the cotranscriptional folding of a downstream secondary structural switch that interfaces with the expression machinery [20]. We had detected the over-expression of CA1 in the synovium of AS patients as compared with samples from RA and OA. It increases the likelihood that elevated CA1 expression in AS synovial membrane may be a consequence of up-regulation in sequence variation of rs34282382 in 5'UTR.

The bone tissue is essentially composed of calcium and phosphate precipitates, and calcium carbonate is only 10% in mean of depositions [21]. Although there is no report about the possible role of CA1 in pathophysiological bone formation processes, CO_3_^2- ^ion was initially regarded as an essential part of the calcium phosphate crystal complex. Biltz *et al*. suggested that the synthesis of bone mineral involves hydrolysis of an initial acidic calcium phosphate precipitate to octacalcium phosphate, which is then converted to octacalcium phosphate carbonate. Octacalcium phosphate carbonate satisfies many criteria for a satisfactory definition of the nature of the bone mineral [22]. Vuola *et al*. implanted blocks of natural coral (calcium carbonate) and its structurally similar derivate in the form of hydroxyapatite (calcium phosphate) in rat latissimus dorsi muscle with autogenous bone marrow to compare their bone-forming capability. They found that bone formation was significantly higher in coral than in hydroxyapatite implants [23]. Ripamonti *et al*. implanted rods and discs (comprising 13% hydroxyapatite and 5% calcium carbonate constructs) into heterotopic rectus abdominis sites and orthotopic calvarial defects of adult non-human primates, respectively. They found that partially converted hydroxyapatite/calcium carbonate constructs induce spontaneous differentiation of bone [24]. The use of natural coral as a bone graft substitute is common in orthopedic surgery. Histological study detected *in vivo *formation of a calcium phosphate-rich layer on the surface of the coral [25]. The above reports indicate that calcium carbonate is involved in initial bone formation, although most mineral deposits are not calcium carbonate but calcium phosphate.

Increased bone resorption is a characteristic of AS and RA [26-29]. It has been found that carbonic anhydrase activity enhanced the stimulation of prostaglandin E_2 _for bone resorption [12]. Some groups found that the carbonic anhydrase inhibitor acetazolamide inhibited bone resorption [30,31]. They suggested that the carbonic anhydrase inhibitors played an antiarthritic role by inhibiting bone resorption. In a case report, we have also reported that methazolamide, an anti-CA drug, may improve AS symptoms [32]. CA1 carries out the catalysis of the following reactions: CO_2 _+ H_2_O = H^+ ^+ HCO_3_^-^. CA1 produces not only HCO_3_^- ^but also hydrogen ion (H^+^). Hydrogen ions are produced in the cytoplasm and are secreted extracellularly by H^+^-ATPase, and the secreted hydrogen ion dissolves bone mineral. Hence, CA1 may contribute to bone resorption in AS and RA by producing hydrogen ions, although the detailed mechanism of CA1 in the processes is still unknown. In RA, focal bone loss is due to excess bone resorption by osteoclasts. In contrast to RA, inflammation in AS often results in excess periosteal bone formation [33]. The present study demonstrated that CA1 had different SNPs associated with RA and AS, supporting that CA1 plays different roles for bone resorption and bone formation by mediating calcification.

## Conclusions

In summary, the present study provided direct data to support involvement of CA1 in calcification, a key step for bone formation and bone remodeling. SNPs in the CA1 encoding gene have a strong association with AS and RA. The above finding may be helpful in understanding the pathogenic mechanism of bone development in RA and AS.

## Abbreviations

AA: ascorbic acid; ALP: alkaline phosphatase; ANOVA: analysis of variance; AR-S: alizarin red; AS: ankylosing spondylitis; Β-GP: β-glycerophosphate; bp: base pair; BSP: bone sialoprotein; CA1: carbonic anhydrase I; CCP: cyclic citrullinated peptide; CI: confidence interval; Ct: comparative threshold cycle; DMSO: dimethyl sulfoxide; HWE: Hardy-Weinberg equilibrium; IRES: internal ribosome entry site; LD: linkage disequilibrium; OA: osteoarthritis; OCN: osteocalcin; OM: osteogenic medium; OR: odds ratio; OSX: osterix; PBS: phosphate-buffered saline; PCR: polymerase chain reaction; RA: rheumatoid arthritis; RF: rheumatoid factor; Runx2: runt-related transcription factor-2; SNP: single nucleotide polymorphism; UTR: untranslated region.

## Competing interests

The authors declare that they have no competing interests.

## Authors' contributions

XC designed and executed the study and prepared the manuscript. YZ and LW performed cell culture, western blotting and real time PCR. YX and JP performed the genotyping. XY collected tissue samples. All authors have read and approved the final manuscript for publication.

## Supplementary Material

Additional file 1 **Genotyping result of Illumina SNP VeraCode microarray**. Genotyping was performed using Illumina VeraCode microarray in a case-control study including 51 ankylosing spondylitis (AS) patients, 267 rheumatoid arthritis (RA) patients and 160 health controls. Click here for file
